# Imaging Nucleation and Propagation of Pinned Domains in Few-Layer Fe_5–*x*_GeTe_2_

**DOI:** 10.1021/acsnano.3c03825

**Published:** 2023-08-29

**Authors:** Michael Högen, Ryuji Fujita, Anthony K. C. Tan, Alexandra Geim, Michael Pitts, Zhengxian Li, Yanfeng Guo, Lucio Stefan, Thorsten Hesjedal, Mete Atatüre

**Affiliations:** † Cavendish Laboratory, Department of Physics, University of Cambridge, Cambridge, CB3 0HE, United Kingdom; ‡ Clarendon Laboratory, Department of Physics, University of Oxford, Oxford, OX1 3PU, United Kingdom; ¶ Department of Physics, Imperial College, London, SW7 2AZ, United Kingdom; § School of Physical Science and Technology, ShanghaiTech University, Shanghai 201210, China; ∥ Center for Hybrid Quantum Networks (Hy-Q), Niels Bohr Institute, 2100 Copenhagen, Denmark

**Keywords:** two-dimensional material, Fe_5_GeTe_2_, nitrogen-vacancy center, quantum imaging, van der Waals materials

## Abstract

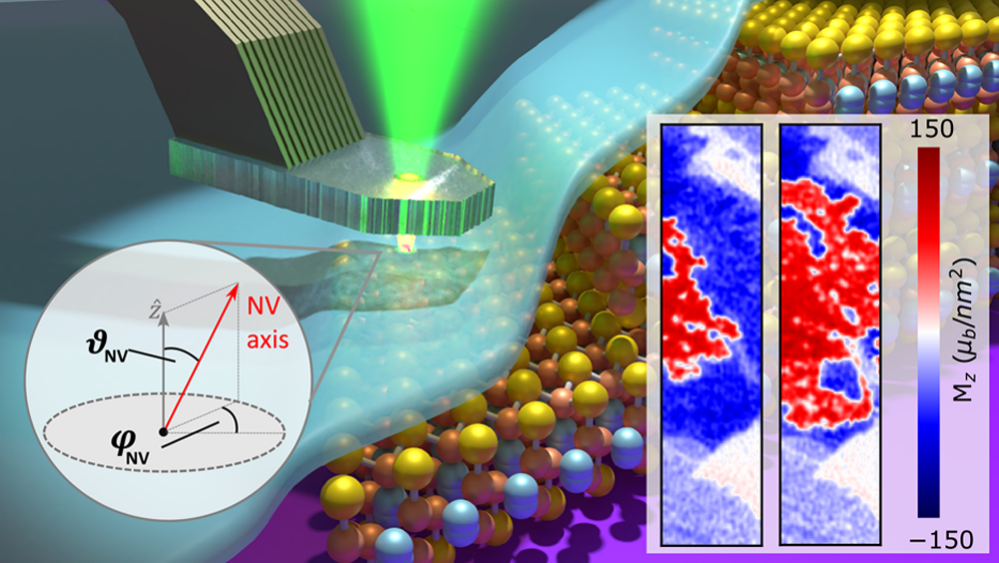

Engineering nontrivial spin textures in magnetic van der Waals materials is highly desirable for spintronic applications based on hybrid heterostructures. The recent observation of labyrinth and bubble domains in the near room-temperature ferromagnet Fe_5–*x*_GeTe_2_ down to a bilayer thickness was thus a significant advancement toward van der Waals-based many-body physics. However, the physical mechanism responsible for stabilizing these domains remains unclear and requires further investigation. Here, we combine cryogenic scanning diamond quantum magnetometry and field reversal techniques to elucidate the high-field propagation and nucleation of bubble domains in trilayer Fe_5–*x*_GeTe_2_. We provide evidence of pinning-induced nucleation of magnetic bubbles and further show an unexpectedly high layer-dependent coercive field. These measurements can be easily extended to a wide range of magnetic materials to provide valuable nanoscale insight into domain processes critical for spintronic applications.

## Introduction

Among the family of itinerant van der Waals (vdW) ferromagnets, Fe_*x*_GeTe_2_ was one of the first materials in which persistent long-range magnetic order down to the monolayer limit was demonstrated. In particular, Fe_3_GeTe_2_ possesses a large perpendicular magnetic anisotropy (PMA) and a high Curie temperature of *T*_C_ = 230 K in bulk^1^ and 130 K in the monolayer limit.^2^ Naturally, this makes it an attractive candidate for hybrid vdW-based heterostructures promising unexplored physical phenomena and functionalities.^3^ For example, Fe_3_GeTe_2_ is currently being intensively explored for next-generation spintronics devices such as magnetic tunnel junctions,^4^ magnetic random access memory technology,^5^ and skyrmionic devices.^6^ Driven by these prospects, material research has in parallel focused on the structurally more complex iron-rich Fe_5_GeTe_2_ compound, which promises richer physical properties and a higher *T*_C_.^7−9^ In particular, the observation of nontrivial spin textures in few-layer thick samples of this recently synthesized material is a significant and exciting development.^10−13^ One of the immediate areas of interest is gaining a better understanding of the physical processes underlying the nucleation of nontrivial spin textures. However, studying two-dimensional (2D) magnets remains challenging due to the restricted pool of techniques capable of spatially exploring spin textures with high sensitivity, nanometer-scale spatial resolution, and with little to no perturbation of the probed magnetization. Moreover, specifically for the study of Fe_5_GeTe_2_ a large applied field is additionally required, which further limits the choice of imaging technique.

In this work, we combine the high magnetic sensitivity and spatial resolution of scanning diamond quantum microscopy (DQM) with well-established field reversal magnetometry techniques to investigate the physical mechanism behind the stabilization of magnetic bubbles in few-layer Fe_5_GeTe_2_. This approach allows us to quantitatively study 2D magnets with high spatial resolution in combination with large out-of-plane (OOP) fields, which would normally be inaccessible to DQM or any other established technique. In particular, by using an AC demagnetization protocol, we reveal surprisingly large coercive fields, *H*_c_, of ∼1.5 T for trilayer and ∼2.2 T for bilayer Fe_5_GeTe_2_. Incorporating first-order field reversal protocols further provides a means of visualizing the high-field formation and propagation of domains. This allows us to unequivocally attribute the presence of strong domain pinning as the main driver of bubble domain formation and the observed strong coercivity in few-layer Fe_5_GeTe_2_.

## Material and Sample Details

**Figure 1:**
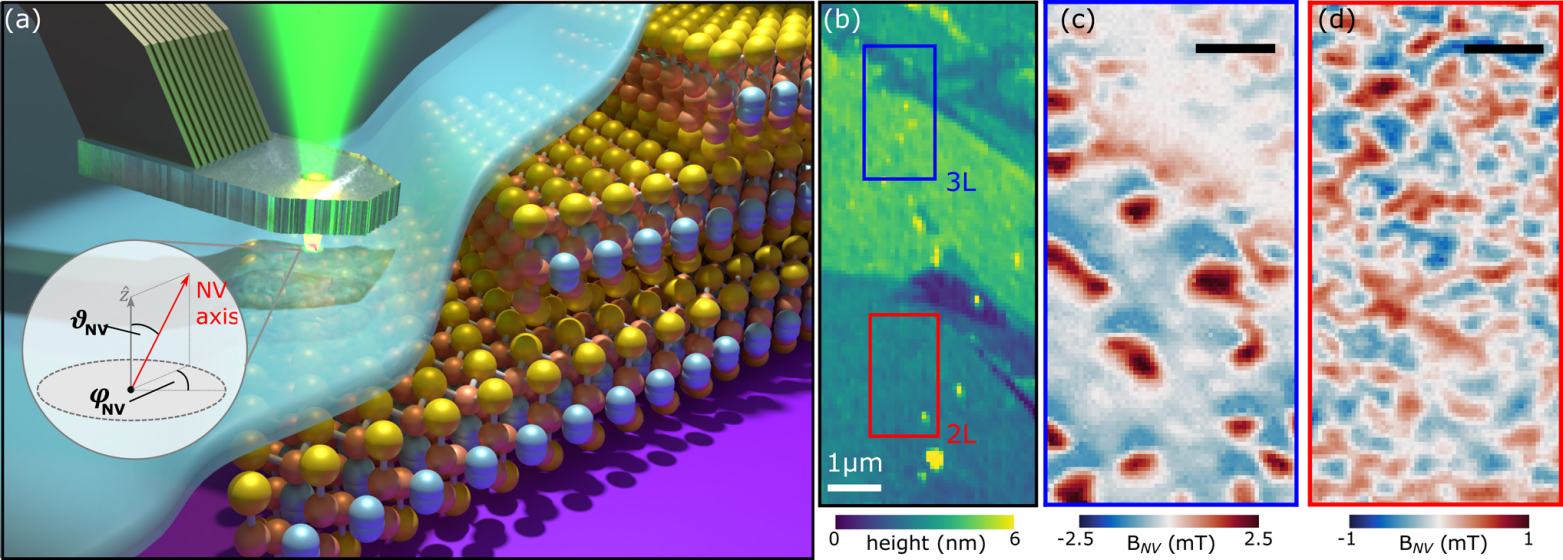
DQM imaging of the few-layer Fe_**5**_GeTe_2_. (a) Schematic (not to scale) of the diamond scanning probe above 1–3 monolayers of Fe_5_GeTe_2_, covered with a thin layer of hexagonal boron nitride (hBN) to prevent oxidation. (b) Topography of the 2L and 3L regions of interest obtained with standard AFM before covering with hBN. (c) Stray field map of the 3L region outlined in blue in (b) after zero-field cooling (ZFC), displaying well-separated magnetic bubble domains. (d) Stray field map of the 2L region outlined in red in (b) after ZFC, showing more random and densely packed magnetic domains. The scale bars in (c) and (d) are 500 nm.

Fe_5_GeTe_2_ crystallizes in a rhombohedral structure (*R*3̅*m* space group), with an arrangement of two-dimensional Fe and Ge slabs sandwiched between Te layers akin to the more familiar Fe_3_GeTe_2_, however, with a more complex local environment.^7^Figure 1a illustrates the Fe_5_GeTe_2_ sample structure representative of the few layer (mono- to trilayer) flakes used in this work (details in Supporting Information (SI)). A single layer consists of an Fe_5_Ge slab (orange and blue balls) sandwiched by two Te layers (yellow balls).^8,9^ The exact stoichiometry can differ, and thus, the candidate Fe_5_GeTe_2_ material is generally referred to as Fe_5–*x*_GeTe_2_. Compared to Fe_3_GeTe_2_, additional complexity can arise in Fe_5_GeTe_2_ due to the presence of vacancy disorder and nonequivalent Fe split-sites, including Fe(1), Fe(2), and Fe(3), where the Fe(1) site typically has an occupation of <50%.^7,8^ Additionally, variations in Fe content, as well as doping with Co, have shown to affect the bulk *T*_C_ (raising it to 270–310 K).^14^ In bulk form, isothermal magnetization data suggest that the magnetic moments align with the *c*-axis, making Fe_5_GeTe_2_ a weak PMA material.^15^ This is in contrast to the few layer limit, where recent magnetotransport measurements indicate that PMA as well as coercivity increase with decreasing layer thickness.

In this study, we employ a Au-assisted mechanical exfoliation process to obtain one to few-layer thick flakes from chemical vapor transport-grown Fe_5_GeTe_2_ bulk crystals.^15,16^ We determined the number of Fe_5_GeTe_2_ layers via atomic force microscopy (AFM); the topography of the area studied is shown in Figure 1b. The two highlighted areas in Figure 1b each correspond to two layers (2L) and three layers (3L) (details in SI), with a monolayer thickness of ∼1 nm. To prevent oxidation of the sample, an amorphous Se capping layer of 5 nm thickness was thermally evaporated on top of the exfoliated Fe_5_GeTe_2_ flake. Next, an ∼20 nm thick hexagonal boron nitride (hBN) layer was placed on top (blue draping layer in Figure 1a) before placing a 20 μm thick copper wire within a 100 μm distance to the flake for supplying the microwave signal to the NV. We then cooled the sample to a base temperature of 4 K in the absence of external magnetic fields.

We use a custom-built cryogenic scanning diamond quantum microscope (DQM)^17^ to image the domains present in bilayer and trilayer Fe_5_GeTe_2_. A diamond probe containing a single nitrogen-vacancy center (NV) at the apex is raster-scanned at a constant height across the sample surface. The NV has an optically addressable spin and thus acts as an atom-sized magnetometer. In the weak field regime, the NV’s spin sublevels *m*_s_ = ±1 linearly shift by 28 MHz/mT with a magnetic field along the NV axis (*B*_NV_).^18^ This Zeeman shift is read out via a pulsed optically detected magnetic resonance (ODMR) protocol, allowing the local stray field distribution 80 nm above the surface to be mapped out. From the stray field maps, we reconstruct the OOP magnetization based on a Fourier method for a two-dimensional (2D), OOP magnetization^19^ (details in SI). The high magnetic sensitivity and spatial resolution make scanning DQM well-suited for nanometer scale studies of 2D magnetism,^20,21^ including twisted CrI_3_.^22^ Such DQM studies are, however, limited to magnets with small *H*_c_ as imaging under large magnetic fields remains challenging. On the one hand, the NV-axis in standard commercial probes forms a 54.7° angle from the OOP axis, thus limiting the ability to perform quantitative imaging in applied fields up to only 10 mT.^23^ On the other hand, even with state-of-the-art probes with an NV-axis aligned along the OOP axis, the efficient delivery of a driving microwave signal to the NV beyond 10 GHz, required for large applied fields, can be challenging. Here, we combine DQM with well-established magnetometry techniques to overcome these limitations, enabling us to quantitatively study few layers of Fe_5_GeTe_2_ with large *H*_c_ values ranging from 1 to 2 T. For all experiments, a small bias field was applied during the measurement to split the degenerate *m*_s_ = ±1 levels in order to determine the direction of the magnetic field. The bias field is weak enough (∼2.5 mT) to not disturb the sample magnetization, while still operating the NV in the weak-field approximation.

## Results and Discussion

### Zero-Field Cooling and Layer-Dependent Magnetic Domains

The Fe_5_GeTe_2_ sample was first cooled down to ∼6 K in zero-field, and DQM was performed above regions with 2L and 3L thickness as outlined in red and blue in Figure 1b. Figure 1c and 1d show the *B*_NV_ distributions corresponding to 3L and 2L Fe_5_GeTe_2_. *B*_NV_ is defined as the local magnetic field projected onto the NV axis, which is at an oblique angle with the OOP direction (details in SI). The field distributions in Figures 1c and 1d reveal an underlying isolated bubble and labyrinth domain pattern, respectively, which are consistent with previous reports on 3L and 2L Fe_5_GeTe_2_.^24^ The average sizes of the observed bubble domains of 400 to 600 nm, and labyrinth domains of 100 to 300 nm are also in agreement. Note that the variation in bubble size hints toward their formation being likely due to defects.

### AC Demagnetization of 2L and 3L Fe_5_GeTe_2_

**Figure 2:**
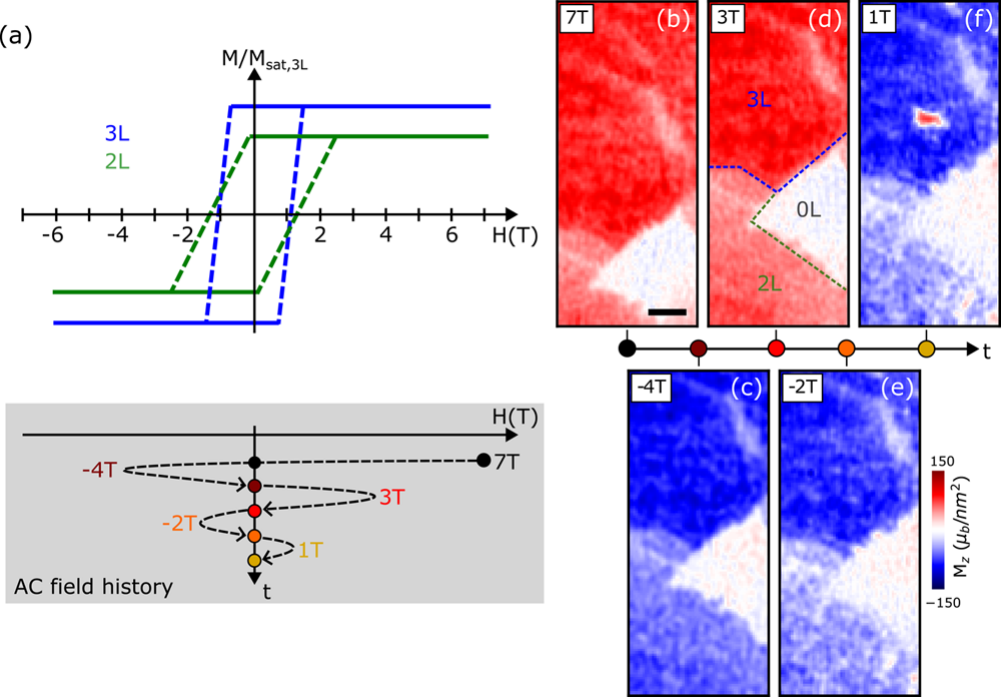
AC demagnetization of few-layer Fe_5_GeTe_2_. (a) Estimated hysteresis loop for 3L (blue) and 2L (green) Fe_5_GeTe_2_ obtained from the AC demagnetization protocol, as sketched below in the gray AC field history box. Starting from saturating with a high external magnetic field of 7 T, the layers are demagnetized by consecutively applying fields of decreasing magnitude with alternating polarity. The remanent state of 2L and 3L Fe_5_GeTe_2_ is imaged after each demagnetization field as shown in (b) to (e). (b–e) Remanent OOP magnetization maps of 3L and 2L Fe_5_GeTe_2_ as a function of demagnetizing field. The scale bar is 500 nm.

We begin the investigation of the mechanism underpinning the observed domains by quantifying the layer number dependence of the OOP saturation field *H*_s_. To this end, we focus on the area between the blue and red boxes in Figure 1b to simultaneously extract information about 2L and 3L Fe_5_GeTe_2_. The *H*_s_ of Fe_5_GeTe_2_ is significantly higher (>1 T) than that of most few-layer vdW ferromagnets studied so far, and applying such a strong OOP field is incompatible with simultaneous NV imaging.^25−27^ While an external field aligned with the NV axis at ∼55° enables optimal imaging by avoiding spin mixing, it is not clear how IP fields would perturb the sample. Moreover, due to the limitations of our setup, including an external field limited to OOP and a microwave delivery not optimized for larger than 10 GHz, quantitative imaging is therefore done at near remanence. Few-layer Fe_5_GeTe_2_ has a large PMA, resulting in the characteristic *M*-*H* loop of a hard magnet,^25,27^ as illustrated in Figure 2a. Since the magnetization at remanence (*H* = 0 T) is still saturated, we can utilize an AC demagnetization protocol to decouple the field application and NV imaging to estimate *H*_s_. The protocol starts with *H*_AC_ = +7 T to saturate the sample, followed by a sequence of decreasing |*H*_AC_| with alternating polarity, as illustrated in the inset of Figure 2a. When |*H*_AC_| reduces to <*H*_s_, domain nucleation is observed. To capture this, DQM imaging is performed at every zero-field crossing of the AC demagnetization sequence (colored dots in Figure 2a insert). The remanent OOP magnetization *M*_*z*_ after each *H*_AC_ step, reconstructed via reverse-propagation (details in SI), is presented in Figure 2b–e.

Figure 2b–d reveals that both the 2L and 3L regions remain saturated down to |*H*_AC_| = 3 T. The onset of domain nucleation in 2L occurs beyond |*H*_AC_| = 2 T (Figure 2e). This is inferred by the appearance of patches of reduced |*M*_*z*_| in the 2L region compared to Figure 2c–d (−4 and 3 T). For 3L, this happens at 1 T (Figure 2f), indicated by the presence of an *M*_*z*_ domain with opposite polarity. We therefore estimate 2 T < *H*_s_ < 3 T for 2L and 1 T < *H*_*s*_ < 2 T for 3L Fe_5_GeTe_2_. Additionally, from the saturated state in Figure 2b–d, we extract an average saturation magnetization of (67 ± 2.5) μ_B_/nm^2^ and (37 ± 1.3) μ_B_/nm^2^ for 3L and 2L Fe_5_GeTe_2_, respectively (see details in SI). Considering the defective nature of Fe_5_GeTe_2_ and the resulting discrepancy between calculations and experimental data, these values are at the lower end of the expected range.^7,28^

### Domain Pinning in 3L Fe_5_GeTe_2_

**Figure 3:**
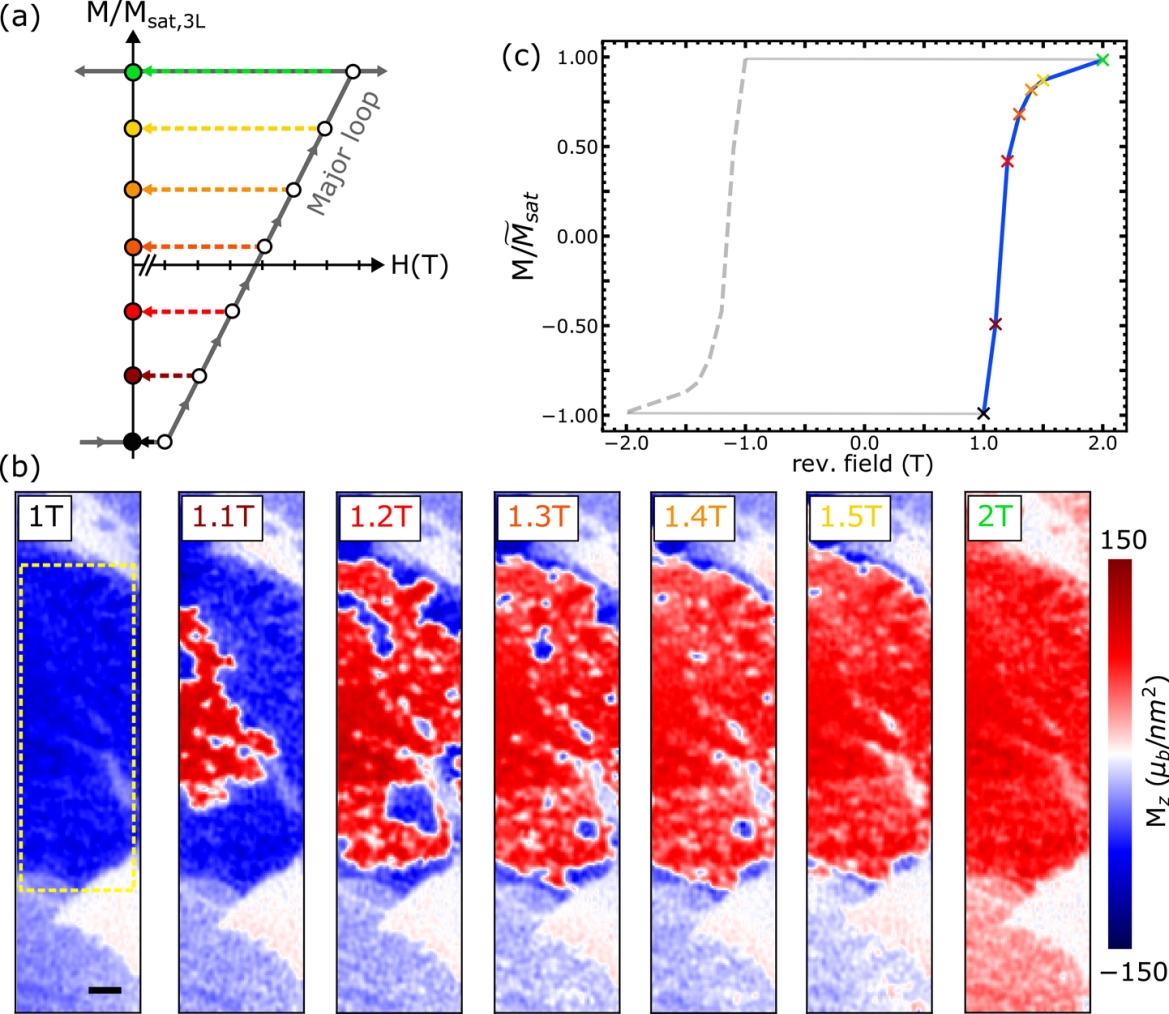
Domain pinning in 3L Fe_5_GeTe_2_. (a) Schematic of the field reversal sequence used to obtain the OOP magnetization maps in (b). For each scan, the sample is saturated at −3 T and subsequently the reversed target field applied (white circles on the major loop), after which the sample is imaged at remanence (colored circles at *H* = 0). For simplicity, the colored dashed lines, indicating the return to the remanent state, are drawn horizontally. In practice, the magnetization of the remanent state is lower than the in-field value. (b) Magnetization maps of the remanent state after reversing to the field displayed in the respective box. A positive domain starts nucleating after reversing to 1.1 T and spreads with increasing reversal field, while small negative domains reappear in the same location after each saturation cycle and stay pinned even at high reversal fields. The scale bar is 500 nm. (c) Pseudohysteresis loop extracted from the OOP magnetization maps in (b). The area considered is outlined by the dashed yellow line in the 1 T scan. The colored crosses correspond to the remanent magnetization after each reversal field, and the dashed line is obtained by mirroring the measured positive field data (i.e., assuming a symmetric hysteresis loop).

Next, we turn to the isolated bubble domain morphology in 3L Fe_5_GeTe_2_ to investigate its propagation and formation mechanism. To this end, we apply a truncated first-order reversal field protocol to the sample,^29−31^ as illustrated in Figure 3a. The field protocol crucially gives DQM access to irreversible magnetization processes occurring at high field, including domain nucleation and propagation,^29−31^ that would otherwise be unfeasible. Our reversal field sequence begins by ramping from saturation at −3 T, as identified previously, to a positive reversal field *H*_r_ (white circles in Figure 3a) and back again to zero for DQM imaging (colored circles). We repeat this with increasing *H*_r_ values, where the magnetization at the point of field-reversal traces out the major *M*-*H* loop (white circles). Figure 3b shows the remanent *M*_*z*_ distribution retrieved with increasing *H*_r_. As anticipated, it reveals a positive *M*_*z*_ domain nucleation at *H*_r_ = 1.1 T and its propagation toward the edges of the magnet at higher *H*_*r*_ until saturation is reached at *H*_r_ = 2 T. Interestingly, the domain propagation is not uniform but instead appears to encircle negative *M*_*z*_ domains of various sizes, similar to the zero-field-cooled bubble morphology in Figure 1c. As *H*_r_ increases, these domains either shrink or undergo fission into smaller domains, which eventually switch to positive *M*_*z*_ at saturation. Further, it is observed that positive *M*_*z*_ domain nucleation and propagation, and the encircled negative *M*_*z*_ domains, occur in approximately the same location on the flake even after undergoing a state reset via saturation after every field sequence with a different *H*_r_. This strongly suggests that the energy landscape is dominated by domain pinning.

The up-sweep of an *M*-*H* loop can be retrieved by extracting the average magnetization over an area at each *H*_r_ from Figure 3b. The average magnetization is defined as *M*/*M̃*_sat_ = (*P*_+_ – *P*_–_)/(*P*_+_ + *P*_–_), where *M̃*_sat_ is the pseudosaturation magnetization at remanence and *P*_+_ and *P*_–_ are the number of pixels corresponding to a positive and negative absolute magnetization value larger than 10 μ_B_/nm^2^, respectively. Figure 3c shows the pseudo *M*-*H* loop over the 3L region (yellow box in Figure 3b) consisting of the retrieved upsweep (blue curve), which is then rotated 180° upon the origin to produce the downsweep (dotted curve). The *M*-*H* loop is consistent with anomalous Hall measurements of flakes of very similar thicknesses.^25,27^ Such macroscopic measurements can thus be microscopically understood by an initially large *H* dependence (i.e., large change in *M* or Hall voltage) due to domain nucleation and fast propagation, followed by a reduced dependence at higher *H* due to domain pinning. A similar study of 2L Fe_5_GeTe_2_ proved to be challenging due to a combination of highly dense domains and an insufficient applied field resolution. However, the established higher *H*_*s*_ of 3 T compared to 3L via AC demagnetization hints toward it being either more affected by pinning or having more pinning sites. Nevertheless, the apparent difference in nucleation characteristics between 2L and 3L Fe_5_GeTe_2_ is consistent with results obtained from XPEEM measurements.^24^ Compared to the single domain switching behavior in 3L, heavily affected by pinning, the nucleation behavior in 2L Fe_5_GeTe_2_ is more complex as the domain frequency increases, indicating a decrease in magnetostatic energy for 2L. A recent study on Fe_5_GeTe_2_ bilayers and monolayers observed soft-ferromagnetism-like behavior due to a decrease in PMA.^27^ However, in our case, we are unable to identify the exact physical mechanism responsible for this behavior. The increase in domains could be due to a combination of factors, including a decrease in anisotropy and symmetric exchange as well as an increase in antisymmetric exchange energy. Further studies of 2L with a higher spatial resolution and finer external field control could reveal more insights into the domain nucleation mechanism. Overall, the untypically high coercive field in our 2L and 3L Fe_5_GeTe_2_ samples aligns well with the observation of a high degree of domain pinning within the material, which we believe is the main coercivity mechanism, while the layer-dependence of the coercive field could be attributed to an enhanced shape anisotropy in 2L Fe_5_GeTe_2_, which is not unexpected as the thickness of the material is decreased.

### Domain Wall Analysis

**Figure 4:**
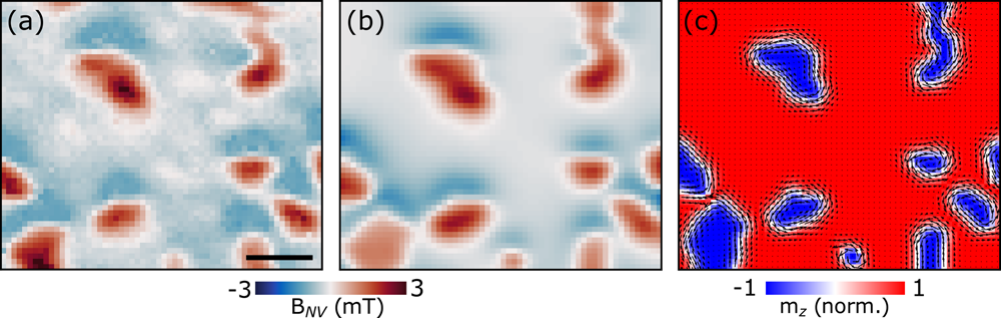
Bubble domain simulation. (a) Close-up of magnetic bubble domains observed after ZFC 3L Fe_5_GeTe_2_. (b) Magnetic field map generated using the domain wall helicity extracted from minimizing the squared spatial residuals between data and simulation. (c) Magnetization distribution consistent with the stray field in (b). The coloring represents the value of the *z*-value of the magnetization, i.e, the OOP magnetization. The best solution suggests almost pure Bloch-type domain walls. Arrows in (c) illustrate the in-plane magnetization components. The scale bar is 400 nm.

While we have established that the domain nucleation in 3L Fe_5_GeTe_2_ is strongly influenced by pinning, there remains a possibility that chiral interactions, such as the interfacial Dzyaloshinskii–Moriya interaction (iDMI), might also play a role. However, as we can conclude that domain walls observed in 3L Fe_5_GeTe_2_ have a Bloch helicity, which is favored in the case of weak or absent iDMI, this scenario is not very likely. Our analysis relies on the distinct stray field character of Bloch and Néel domain walls. While Bloch walls only generate stray fields through the z-component of the magnetization, as ∇⃗ · *M⃗*_*xy*_ = 0, Néel walls do generate additional stray fields through the in-plane components of the magnetization, since ∇⃗ · *M⃗*_*xy*_ ≠ 0, where *M⃗*_*xy*_ is the in-plane magnetization vector (details in SI).^32,33^ We simulate the stray field of the bubble domains in Figure 1c as a function of helicity ξ and compare it with the experimental observations. We use the OOP magnetization obtained via reverse-propagation from Figure 1c as a first approximation to determine areas with up and down domains, from which we also extracted an average domain wall width of ∼135 nm. The OOP magnetization map is then segmented to identify domain boundaries. This allows us to generate a domain map with various wall widths and helicities. Finally, using the saturation magnetization extracted from AC demagnetization, we generate the stray field 80 nm above the surface. By minimizing the sum of squared differences between the experimental and simulated stray fields, we extract the most probable helicity value (details in SI). Figure 4a shows the region of the experimental data used for this analysis. Figure 4b is the stray field map generated using the optimal helicity ξ ≈ π/2, indicating predominantly Bloch-type domain walls in experiments. Finally, Figure 4c shows the optimal Bloch-like winding (ξ ≈ π/2) of the in-plane magnetization (arrows) overlaid with the out-of-plane magnetization component. The presence of Bloch domain walls in Fe_5_GeTe_2_ further supports the stabilization of bubble domains caused by a high degree of pinning rather than chiral interactions such as iDMI.

### Conclusion

The combination of demagnetization and field reversal protocols in cryogenic scanning DQM offers key insights into the high-field-dependent microscopic behavior of hard vdW magnets with high saturation fields. Our multimodal DQM approach has established markedly different domain morphologies between 2L and 3L Fe_5_GeTe_2_. It has crucially revealed a highly pinned energy landscape dominating the domain nucleation and propagation processes, void of any substantial interfacial chiral interactions in few-layer Fe_5_GeTe_2_. Further investigation into the origin of the pinning will be necessary for a full understanding and for incorporating Fe_5_GeTe_2_ into next generation spintronic devices in the future. Our measurements have demonstrated that DQM is well-suited for studying the magnetic energy landscape of two-dimensional materials with emergent properties, including chiral interactions, by discriminating between spontaneous and pinning-assisted nucleation and by extracting their chirality.

## Materials and Methods

### Sample Preparation

Bulk single crystals of Fe_5_GeTe_2_ were grown by chemical vapor transport from an iron-enriched mixture consisting of Fe, Ge, and Te at a 6:1:2 ratio; see SI for bulk characterization. Atomically thin flakes of Fe_5_GeTe_2_ were exfoliated onto Si/SiO_2_ using gold-assisted mechanical exfoliation from the bulk single crystal. A thin amorphous layer of selenium was evaporated to protect the sample from degradation. Subsequently, an additional thin and homogeneous flake of hBN was placed on the Fe_5_GeTe_2_ flake to protect the scanning probe from picking up contamination. Thicknesses were determined by optical contrast and atomic force microscopy. The data in the main text are obtained from a single sample. For more details please see the SI.

### Diamond Quantum Microscopy

The diamond quantum microscope (DQM) is an integrated confocal and atomic force microscope housed in a closed-cycle cryostat (attoDRY1000, Attocube Systems) equipped with a 9 T single-axis superconducting magnet. The confocal optics are home-built, and the atomic force microscope platform is based on an electrically read-out tuning fork. All measurements are conducted at 4 K, unless specified otherwise. The NV center is optically excited and read out using a pulsed-ODMR protocol, which tracks the spin resonances for improved sensitivity and reduces heat build-up. We adopt a pulsed ODMR protocol consisting of a microwave π-pulse and a subsequent laser pulse for combined readout and spin initialization. A wait time of 600 ns before the next microwave pulse ensures relaxation of trapped population toward the ground state. We utilized diamond scanning probes with a single NV center, implanted with an energy of 7 keV, at the apex (QZabre AG) for imaging. The NV-to-sample distance as well as the axis orientation are determined ex-situ via independent calibration measurements (see details in SI). Microwaves for ODMR measurements are delivered via a 20 μm thick copper wire mounted close to the Fe_5_GeTe_2_ flake.

## Supporting Information Available

Section on sample properties and preparation and figure of flakes (optical micrograph), layer-dependent AFM thickness calibration, and magnetic properties; section on the physics and limitations of NV sensing; section on the diamond quantum microscopy setup; section on the characterization of the NV center sensor and figures of the Zeeman splitting of the upper NV spin transition and the Zeeman shift as a function of position; section on reverse propagation and figure on the average magnetization values; section on domain pinning and figure of the pinning probabilities; and finally a section on the domain wall simulation details containing a figure illustrating the domain boundary analysis. (PDF)
