# Supplementary material for: Imaging Nucleation and Propagation of Pinned Domains in Few-Layer Fe5–xGeTe2
Source: ACS Nano. 2023 Aug 29;17(17):16879–85. doi: 10.1021/acsnano.3c03825 (PMC10510720; doi:10.1021/acsnano.3c03825)
Supplement: Supplementary file 1 — nn3c03825_si_001.pdf [file nn3c03825_si_001.pdf]

— Supporting Information for —

Imaging Nucleation and Propagation of Pinned Domains in Few-Layer $Fe_{5\text{-}x}GeTe_2$

Michael Högen,[†,⊥] Ryuji Fujita,[‡,⊥] Anthony K. C. Tan,[†,¶,⊥] Alexandra Geim,[†] Michael Pitts,[†] Zhengxian Li,[§] Yanfeng Guo,[§] Lucio Stefan,[‖] Thorsten Hesjedal,[*,‡] and Mete Atatüre[*,†]

†*Cavendish Laboratory, Department of Physics, University of Cambridge, Cambridge, CB3 0HE, United Kingdom*

‡*Clarendon Laboratory, Department of Physics, University of Oxford, Oxford, OX1 3PU, United Kingdom*

¶*Department of Physics, Imperial College, London, SW7 2AZ, United Kingdom*

§*School of Physical Science and Technology, ShanghaiTech University, Shanghai 201210, China*

‖*Center for Hybrid Quantum Networks (Hy-Q), Niels Bohr Institute, 2100 Copenhagen, Denmark*

⊥*These authors contributed equally to this work*

E-mail: thorsten.hesjedal@physics.ox.ac.uk; ma424@cam.ac.uk

S1 Sample properties and preparation

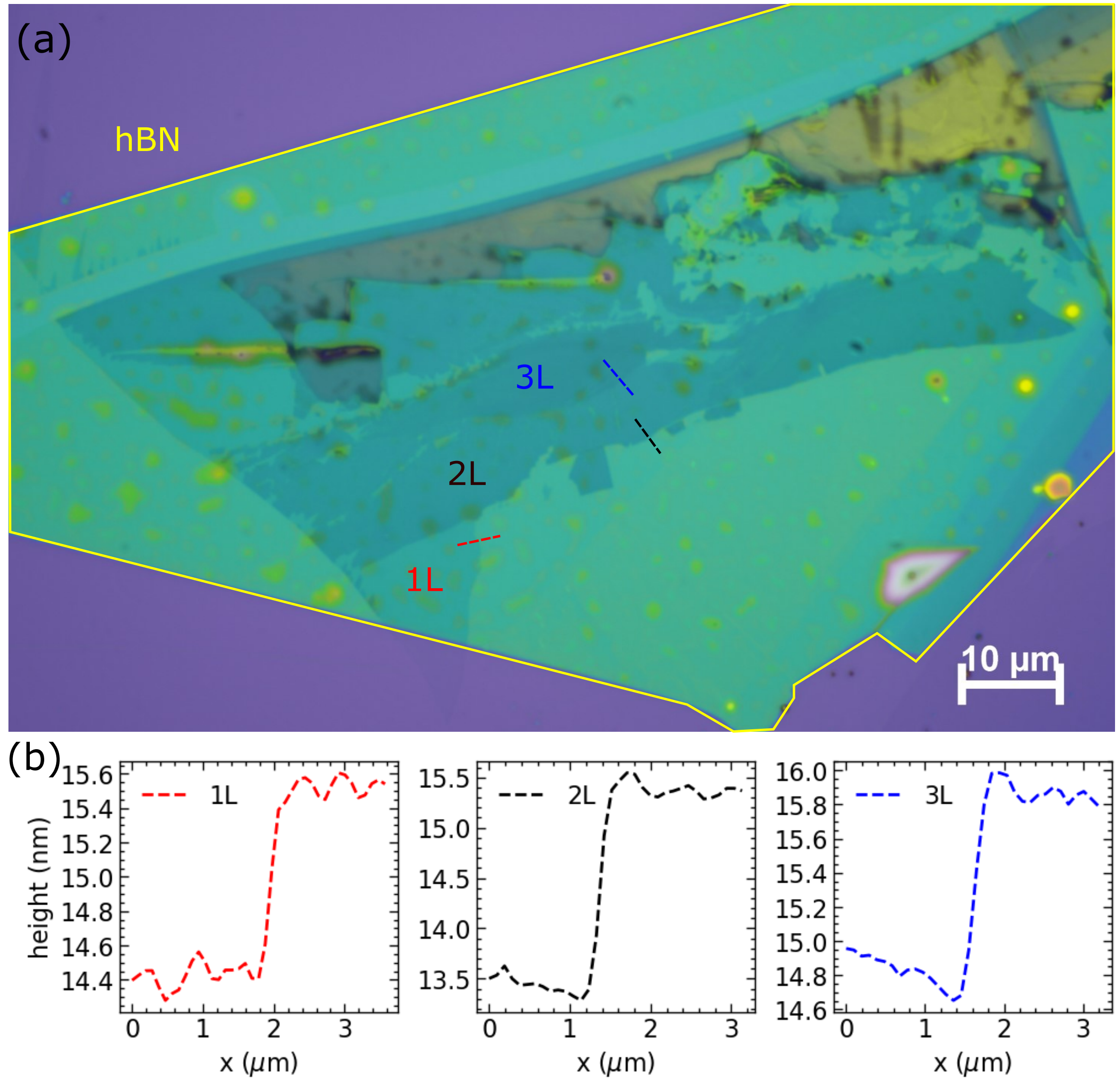

Figure S1: **Sample characterization.** (a) Optical image of the exfoliated flake capped with hexagonal boron nitride (hBN), which is outlined in yellow. (b) AFM line scans across the edges, indicated by the colored dashed lines in (a), confirming the flake thicknesses of 1L, 2L and 3L.

An iron-enriched mixture consisting of Fe, Ge and Te at a 6:1:2 ratio was sealed in an ampule and held at 700° in a tube furnace, yielding single crystals of Fe_5GeTe_2 after slowly cooling down after a few days. An average ratio of Fe:Ge:Te = 4.6:1:2.1 was extracted for the stoichiometry, but it is noteworthy that the stoichiometry differs across the as-grown bulk crystal. The Curie temperature of the bulk crystal was determined to be $T_C = 260\,\mathrm{K}$

using vibrating sample magnetometry (VSM, see Fig. S2). While it is possible to exfoliate Fe_5GeTe_2 down to about ten layers, it becomes increasingly difficult to obtain flakes thinner than that. Here, we exploit the interaction between gold (Au) and the top-most layer of the Fe_5GeTe_2 crystal to exfoliate layers as thin as a monolayer. The sample was originally capped by a thin layer of amorphous selenium (Se) to avoid degradation. From experience, this Se cap typically also results in small islands, which can be moved easily with a scanning probe. To reduce the risk of contaminating the diamond tip with Se, the sample was additionally capped with a 20 nm thick and homogeneous flake of hexagonal boron nitride (hBN), indicated by the yellow outline in Fig. S1(b). In the 2D community, hBN is routinely used as a capping layer to avoid degradation. It is free of dangling bonds and very hard, which makes it an ideal surface for the diamond tip. Keeping the hBN thickness to 20 nm or below ensures that the NV's spatial resolution is not overly compromised.

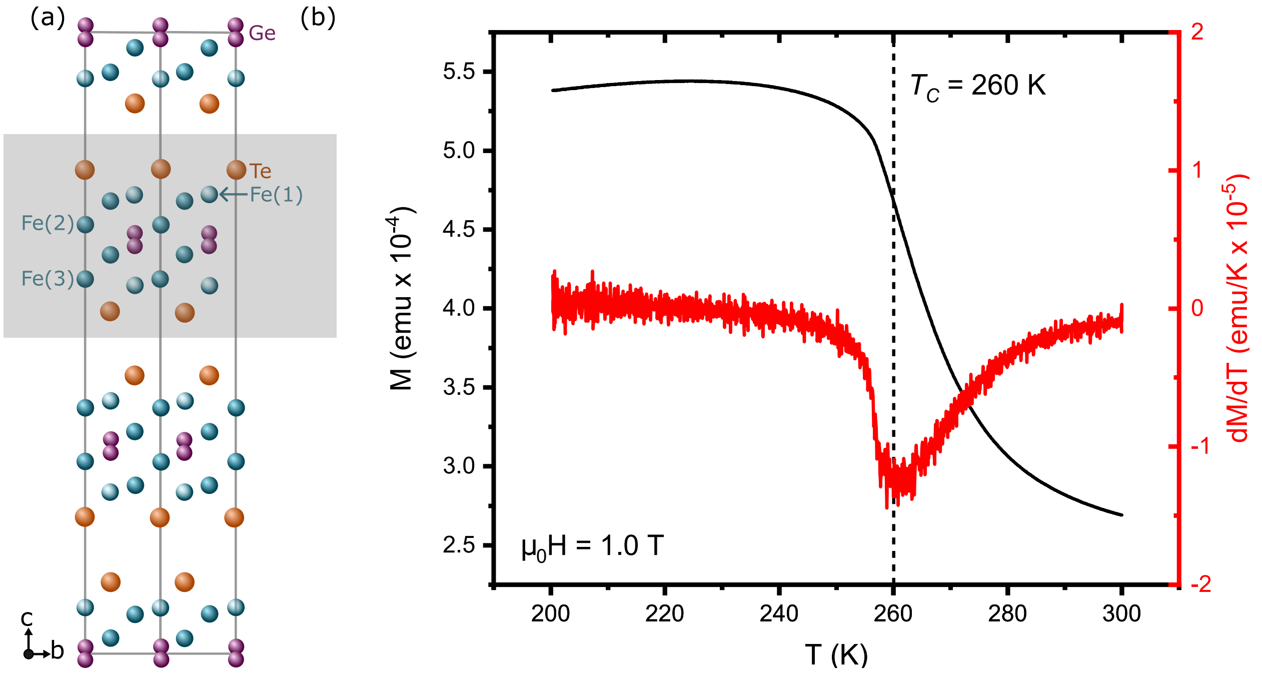

Figure S2: **Crystal strucutre and VSM of bulk Fe_5GeTe_2.** (a) Side view of the average Fe_5GeTe_2 crystal structure with Fe(1) and Ge being split sites. (b) M-vs-T measurement of the bulk Fe_5GeTe_2 crystal using a vibration sample magnetometer, yielding a T_C of 260 K.

S2 Physics and limitations of NV sensing

The NV center is an atomic-sized defect in diamond, consisting of a substitutional nitrogen atom next to a lattice vacancy. The ground state manifold of the NV forms a spin triplet with spin sublevels $m_s = 0, \pm 1$ and the quantization axis (NV axis) along one of the four crystallographic axes of diamond. The crystal field splits the $m_s = 0$ from the $m_s = \pm 1$ by 2.87 GHz. An additional Zeeman splitting between the $m_s = -1$ and $m_s = +1$ levels is induced by an external magnetic field along the NV axis, B_{NV}. In the weak field approximation, the frequency splitting, $\Delta\nu$ is linear and given as $2\gamma B_{NV}$, with γ being the gyromagnetic ratio of 28 MHz/mT. Optical excitation results in a spin-state dependent fluorescence, depending on whether the NV spin population is predominantly in the $m_s = 0$ (bright) or $m_s = \pm 1$ sublevel (dark). Combined with ground state spin control via microwave excitation, one can optically detect the electron spin resonance, which is commonly known as optically detected magnetic resonance (ODMR). In order to retrieve the field polarity, we apply a small bias external field, B_{bias}, along the NV-axis. Specifically, this allows us to extract the frequency splitting $\Delta\nu$ to determine the total local field (including the bias field) projected on the NV-axis, given as $|B_{total}| = B_{NV} + B_{bias}$. The actual field component due to the sample is then given as $B_{NV} = |B_{total}| - B_{bias}$. The stray field maps in the main text are obtained by recording and fitting a full ODMR spectrum at one of the two NV transition frequencies at every pixel of the image. We adopt a pulsed ODMR protocol consisting of a microwave π-pulse and a subsequent laser pulse for combined readout and spin initialization. A wait time of 600 ns before the next microwave pulse ensures relaxation of trapped population towards the ground state.[1]

Imaging based on ODMR is limited to magnetic fields below ~ 10 mT due to spin-mixing induced by the orthogonal component of the magnetic field. This results in an inefficient spin polarization that leads to a decreased NV fluorescence as well as ODMR contrast.[2,3] Standard diamond probes typically have NV centers pointing at an oblique angle with respect to the out-of-plane z-axis, which renders the study of few-layer van der Waals magnets with

strong perpendicular magnetic anisotropy (PMA) but low moments in a high out-of-plane (OOP) field environment extremely challenging. While prototype diamond probes with NV centers parallel to the z-axis are now available and could prove useful,[4] imaging at Tesla fields is still challenging from an instrumentation point of view. The microwave frequencies required for ODMR very quickly approach hundreds of GHz, requiring special hardware and microwave delivery solutions to the NV.

S3 Diamond quantum microscopy setup

The diamond quantum microscope (DQM) is an integrated confocal and atomic force microscope housed in a closed-cycle cryostat (attoDRY1000, Attocube Systems). The confocal optics is home-built and the atomic force microscope platform is based on an electrically read-out tuning fork. All measurements are conducted at 4 K, unless specified otherwise. The NV center is optically excited and read out using a pulsed-ODMR protocol which tracks the spin resonances for improved sensitivity and reduces heat build-up. We utilize diamond scanning probes with a single NV center, implanted with an energy of 7 keV, at the apex (QZabre AG) for imaging. Microwaves for ODMR measurements are delivered via a 20 µm thick copper wire mounted close to the Fe_5GeTe_2 flake. More details of the setup can be found in Ref.[5]

S4 Sensor characterization

S4.1 NV axis orientation

The orientation of the NV axis (the axis joining the vacancy and the nitrogen atom) with reference to the laboratory frame is characterized by monitoring the Zeeman splitting of the spin resonances as a function of applied magnetic field $\vec{B}\,(B_0, \vartheta_B, \varphi_B)$, using a three-axis Helmholtz coil. The maximum Zeeman splitting occurs when the field is parallel to the NV

axis. Holding the field at a constant magnitude, we obtain ODMR spectra while varying the polar ϑ_B (azimuthal φ_B) as shown in Fig. S3, resulting in $\vartheta_{\mathrm{NV}} = 120° \pm 4°$and $\varphi_{\mathrm{NV}} = 96° \pm 4°$.

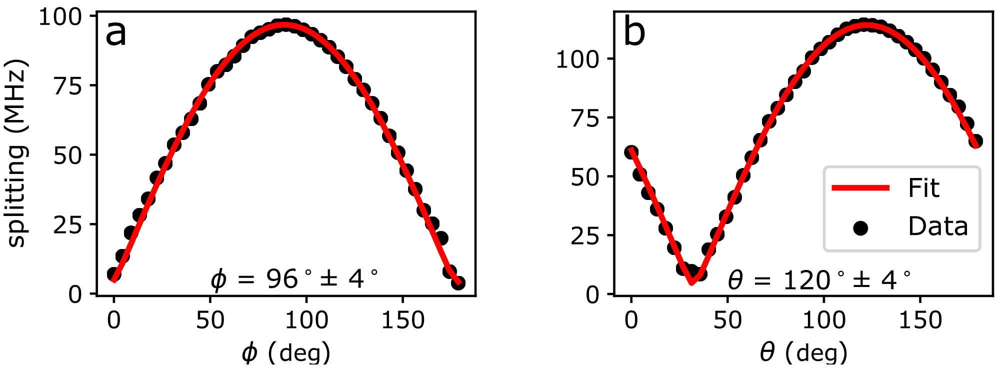

Figure S3: **Zeeman splitting of the upper NV spin transition.** (a) As a function of φ_B with ϑ_B fixed at 90°, and (b) as a function of ϑ_B with φ_B fixed at 96°. The maxima in the curves determine the NV orientation, giving $\vartheta_{\mathrm{NV}} = 120° \pm 4°$and $\varphi_{\mathrm{NV}} = 96° \pm 4°$.

S4.2 NV-to-sample distance

We infer the NV-to-sample distance d_{NV} by measuring the magnetic field produced by the edge of a uniformly magnetized stripe.[6] The calibration sample consists of a film of CoFeB patterned into magnetic wires, which remain saturated at remanence. A single linescan showing the position of the upper spin resonance extracted from ODMR measurements, along with the topography of the edge, is shown in Fig. S4(a). The red curve is a fit to the data, which directly gives the value for d_{NV}. A statistical average value of (60 ± 5) nm is obtained from 20 linescans, as shown in Fig. S4(b). Note that an additional 20 nm needs to be accounted for due to the thickness of the hBN capping layer, yielding a total NV-to-sample distance of $d_{\mathrm{NV}} \sim 80$ nm.

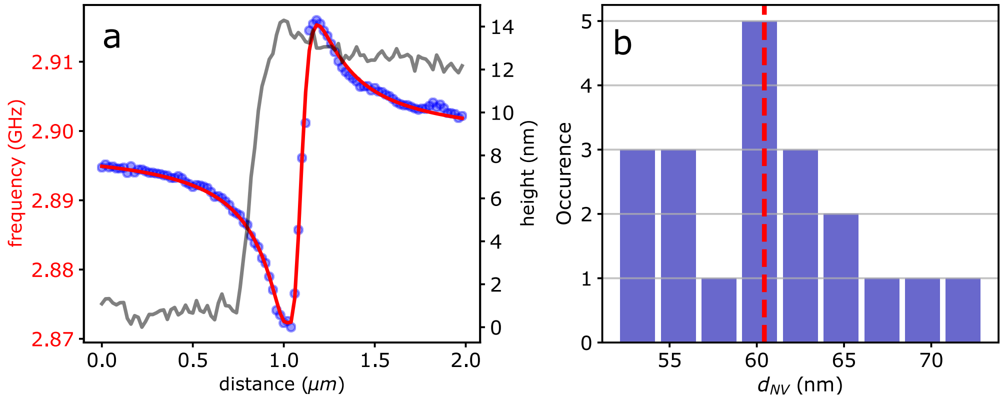

Figure S4: **Zeeman shift of the upper spin resonance as a function of position above the magnetic wire, as indicated by the topographic height of the edge.** An average NV-to-sample distance of (60 ± 5) nm has been extracted from 20 line scans across the edge.

S5 Reverse propagation

The magnetostatic problem of retrieving magnetization from a magnetic field distribution is in general ill-posed. However, in the limit of a thin magnet and a predominantly OOP magnetization (i.e., few layer Fe_5GeTe_2), the problem is sufficiently constrained to produce a unique solution. Assuming a planar magnetization distribution $\mathbf{M}$ that is constant throughout its thickness t, the stray field $\mathbf{B}$ generated at a distance d from the surface is related to the magnetization in Fourier space (k_x, k_y) as:

$$\mathbf{b}(k_x, k_y, z) = \boldsymbol{\mathcal{D}}(k_x, k_z, z)\mathbf{m}(k_x, k_y, z), \tag{S1}$$

with the dipolar tensor given as:

$$\boldsymbol{\mathcal{D}}(k_x, k_z, z) \; = -\frac{\mu_0}{2k^2}\left(e^{-kd} - e^{-k(d+t)}\right)\begin{pmatrix} k_x^2 & k_x k_y & i k_x k \\ k_x k_y & k_y^2 & i k_y k \\ i k_x k & i k_y k & -k^2 \end{pmatrix}. \tag{S2}$$

Here, $\mathbf{b}(k_x, k_y, z)$ and $\mathbf{m}(k_x, k_y, z)$ are the spatial Fourier transforms of $\mathbf{B}(x, y, z)$ and $\mathbf{M}(x, y, z)$, respectively.[7] Indeed, the non-invertible matrix in eq. (S2) is a direct indicator that the pla-

nar magnetization distribution cannot be uniquely reconstructed from the measured stray field. However, if we assume a strong perpendicular magnetic anisotropy in our system, such that $M_{x,y} = 0$ and $M_z \neq 0$, the problem is then well-posed and a unique solution can be found.[8,9] The M_z component can then be directly reconstructed from the z-component of the magnetic field:

$$m_z(k_x, k_y) = \frac{b_z(k_x, k_z)}{\alpha(d_{\mathrm{NV}}, t)}, \tag{S3}$$

with $\alpha = -\frac{\mu_0}{2k^2}\left(e^{-kd} - e^{-k(d+t)}\right)$ and the magnetic field projected on the NV axis with reference to the lab frame can be expressed as

$$B_{\mathrm{NV}}(x, y) = B_x \cos\varphi_{\mathrm{NV}} \sin\vartheta_{\mathrm{NV}} + B_y \sin\varphi_{\mathrm{NV}} \sin\vartheta_{\mathrm{NV}} + B_z \cos\vartheta_{\mathrm{NV}}. \tag{S4}$$

For the reverse propagation of the stray field maps in the main text, an additional Hann filter is used to suppress noise.[8,10] From the reconstructed OOP magnetization, we can determine the saturation magnetization by plotting the flattened magnetization distribution and extract the peak positions of the statistics. Due to sample inhomogeneity and small but finite reconstruction errors, the magnetization values are distributed around the saturation magnetization values and close to zero, corresponding to the domain walls and gaps in the material. The average saturation magnetization values are extracted from the remanent states after saturating in fields of 7 T, −4 T, 4 T and 3 T, as displayed in Fig. S5.

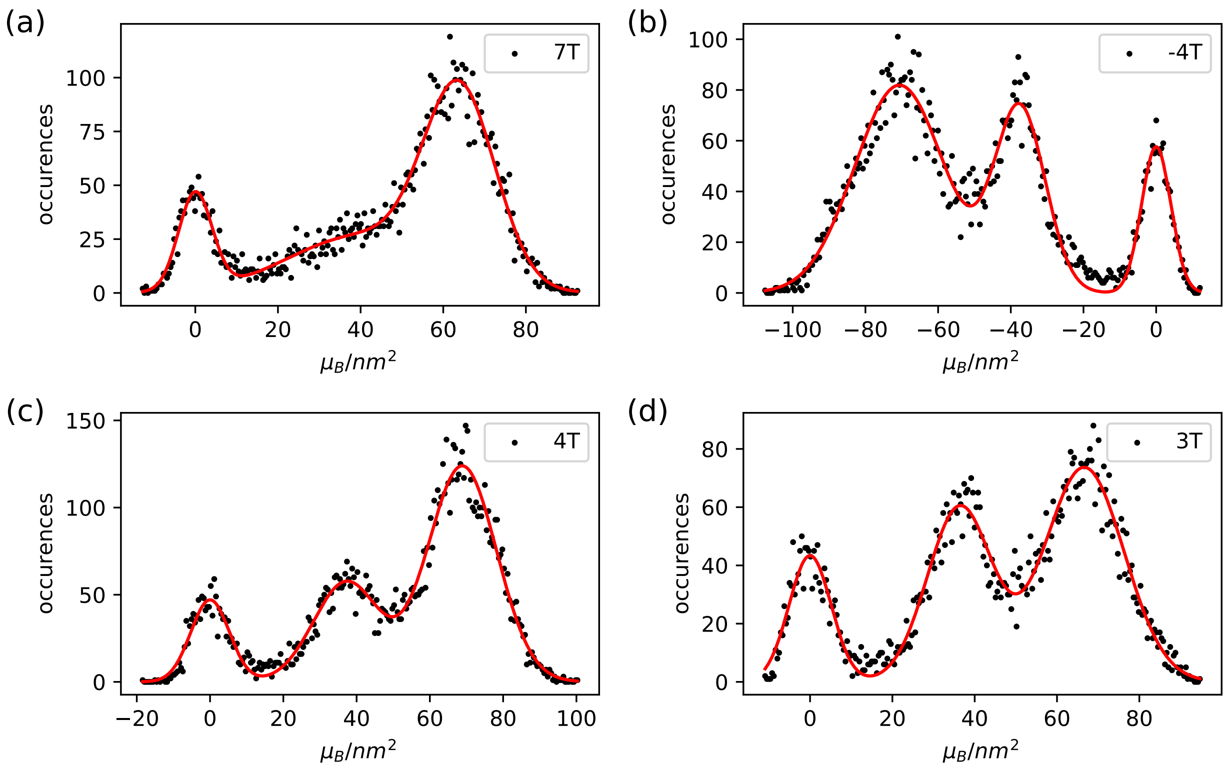

Figure S5: **Reconstructed OOP magnetization distribution of the remanent state.** The panels represent the distribution after saturating with (a) 7 T, (b) −4 T, (c) 4 T and (d) 3 T. The red curves are Gaussian fits to retrieve the peak values.

S6 Size analysis of ZFC morphology

The size of domains in 2L and 3L Fe_5GeTe_2 as observed after zero field cooling, was determined using two separate approaches, respectively. For the bubble morphology in 3L, a thresholded map was separated in regions, each containing a single bubble domain (see Fig. S6(a)). Since the shapes vary drastically between domains, we chose the major axis of each domain as a common denominator, yielding a mean value of 350 nm (Fig. S6(b)). Rather than an absolute size, we extracted a domain period from 2L Fe_5GeTe_2 using a 2D autocorrelation. The autocorrelation map of 2L Fe_5GeTe_2 after ZFC is shown in Fig. S7, including the line profiles to extract the peaks, which in turn correspond to a high degree of similarity. For each line profile, the mean difference between peaks is extracted and plotted

in (c). The mean of these values is indicated by the blue line, yielding a mean period of 390 nm, which approximately translates to a mean domain size of 195 nm.

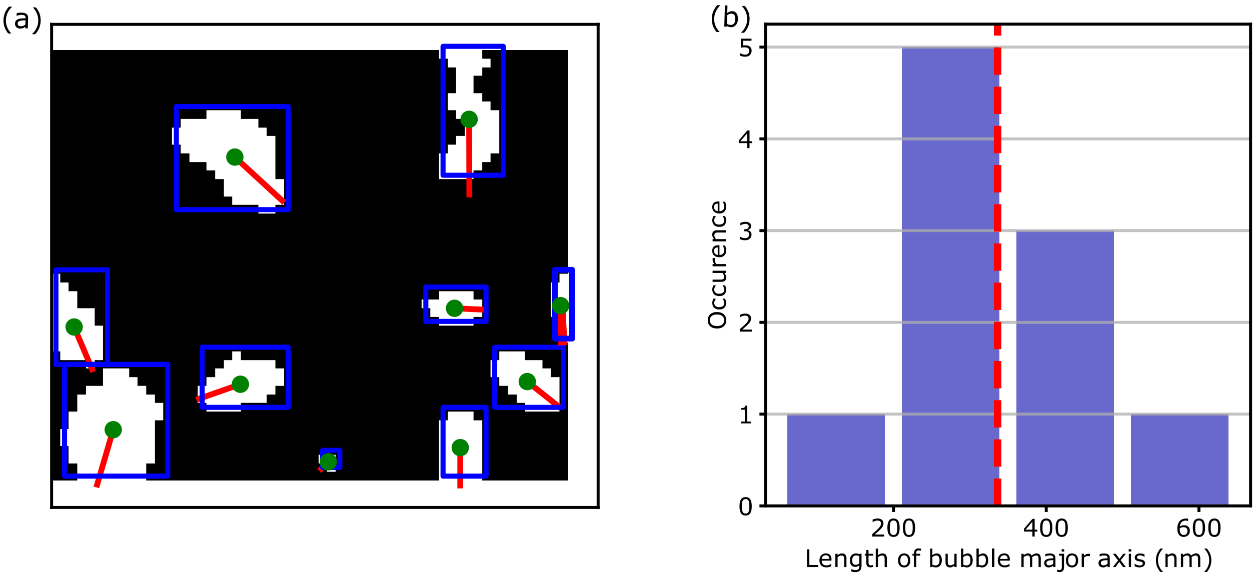

Figure S6: **3L bubble domain size analysis.** (a) Separation of thresholded map into regions, each containing a bubble domain. The red lines indicate half of the major axis. (b) Histogram of values extracted from (a) with a mean value of 350 nm indicated by the red dashed line.

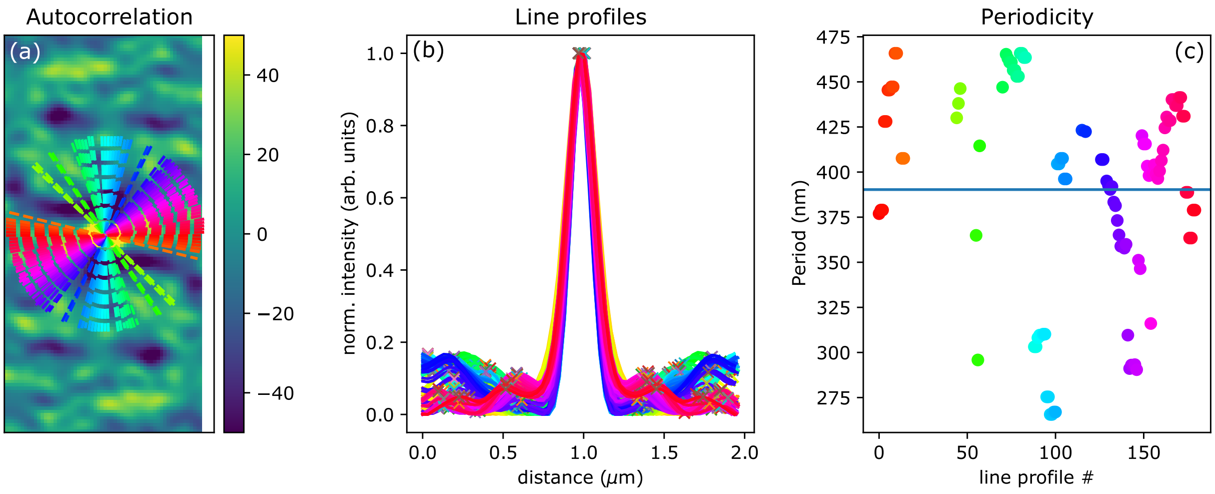

Figure S7: **2L domain analysis.** (a) Autocorrelation map of Fig.1(d) of the main text and line profiles used to extract the peak positions. (b) Autocorrelation line profiles corresponding to the lines in (a). (c) Single values for the domain period extracted from each line profile and the mean indicated by the blue line.

S7 Domain pinning

We visualize the domain pinning observed in Fig. 3(b) of the main text by spatially quantifying the pinning probability. For the specific example of Fig. 3(b), an area is identified as pinned when it remains unpolarized (blue) while its surrounding is polarized (red) by the applied magnetic field. Since the sample undergoes a magnetic reset via a negative saturation field for each of the image in Fig. 3(b), we can repeat the analysis above on every image to collate the total number of times an area gets pinned. The probability of an area to get pinned is then extracted by normalizing the total pinned occurrences by the number of instances the area was identified polarized (red). Figure S6 shows the heatmap of pinning probability over the sampled region in Fig. 3(b). It strongly indicates that high pinned probabilities are correlated with areas of smaller size, and that pinning is distributed across the magnetic material.

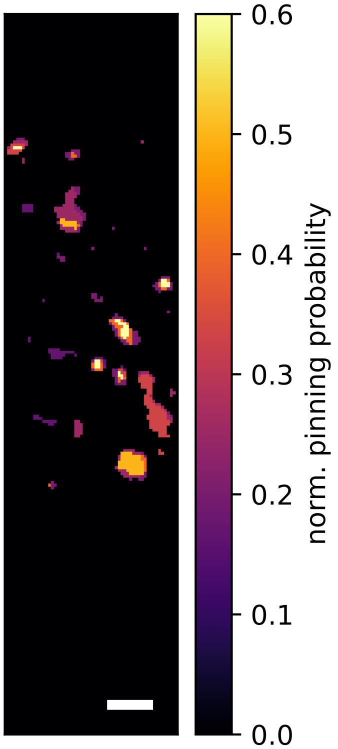

Figure S8: **Heatmap indicating pinning probability of magnetic domains in 3L Fe_5GeTe_2.** The pinning landscape intrinsic to and extracted from figure 3(b). The scale bar is 500 nm.

S8 Details on domain wall simulation

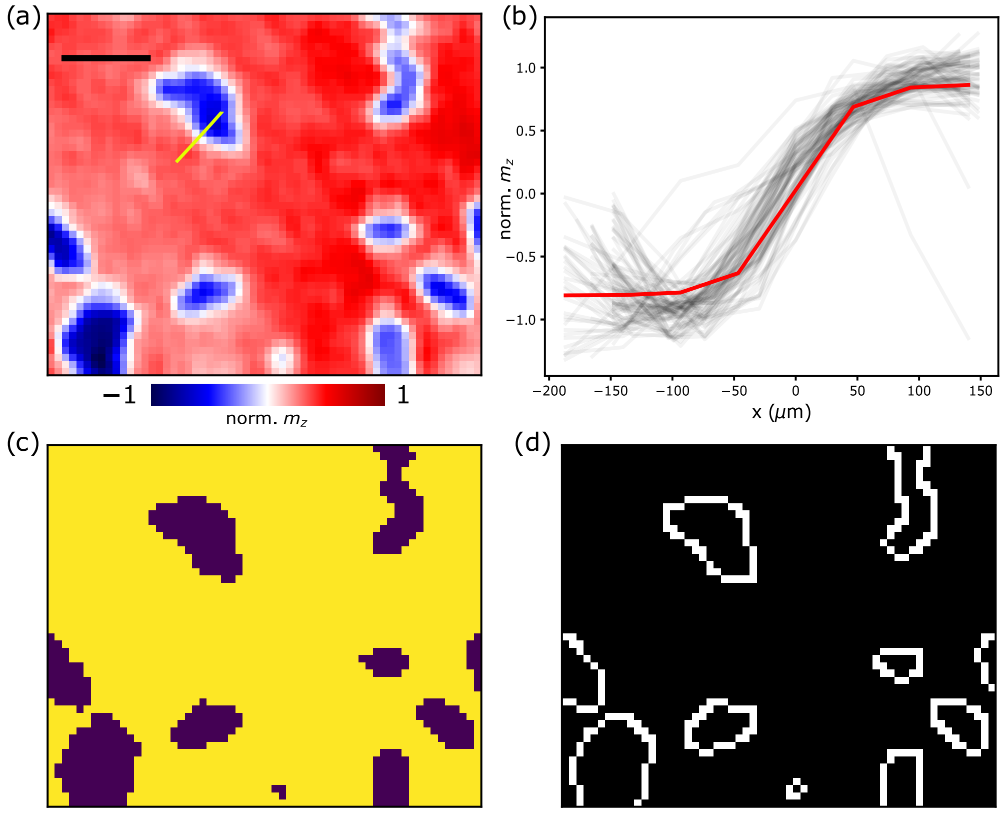

Figure S9: **Domain boundary analysis.** (a) Normalized z-component of the magnetization from reverse propagating the stray field map shown in Fig. 1(c) in the main text. (b) Line profiles extracted from (a) and as exemplified by the yellow line in (a). The fit in red allows to extract the average domain wall width, giving a value of 137 nm. (c) Binary image obtained from Otsu thresholding the map in (a). (d) Domain boundaries found by edge detection.

Here we outline the steps for the simulation of the magnetic bubbles as a function of helicity as well as the minimization of the cost function. Neglecting the contribution of the domain walls to the stray field, we can directly reconstruct M_z from the stray field map in Fig. 1(c) in the main text. The average domain wall width is found by extracting line profiles across all domain walls visible in Fig. S9(a). The average normalized magnetization is then fitted

to the standard analytical description of a domain wall:

$$\begin{aligned} M_x &= \sqrt{1 - M_z^2} \cos(\phi + \xi) \\ M_y &= \sqrt{1 - M_z^2} \sin(\phi + \xi) \\ M_z &= \tanh(\pi(s - s_0)/w) \ . \end{aligned} \tag{S5}$$

Figure S9(b) illustrates extracted line profiles (black line) and the fit to the profile average (red line) which gives a mean domain wall width of ∼137 nm. The map in Fig. S9(a) is then binarized via Otsu thresholding (Fig. S9(c)) and domain boundaries are detected using the Canny edge algorithm (Fig. S9(d)). Finally, the edge normal vectors are found and at every edge pixel, and a domain wall is created along each normal vector, with s_0 being the center of the domain wall. The magnetic field generated at a distance d above the simulated magnetization distribution can be calculated using the dipolar tensor introduced in section S5. We then minimize the sum of the squared differences between the experimentally obtained B_{NV}^{exp} and the simulated B_{NV}^{sim} with respect to the helicity ξ, which can be expressed with a cost function:

$$C = \sum_{N_x, N_y} (B_{\mathrm{NV}}^{exp} - B_{\mathrm{NV}}^{sim})^2 \quad , \tag{S6}$$

where N_x and N_y are the number of pixels in x and y, respectively, while fixing the NV-to-sample distance $d_{\mathrm{NV}} = 80\,\mathrm{nm}$, the domain wall width $w_{\mathrm{DW}} = 137\,\mathrm{nm}$, and the surface magnetization at $I_s = 66\,\mu_{\mathrm{B}}/\mathrm{nm}^2$.

S9 Details on domain wall width extraction

We validate our approach of extracting the domain wall width from bubble domains by simulating m_z with a fixed wall width of 135 nm based on the bubble morphology displayed in the main text and SI sections above (see Fig. S10(a). We extract a domain wall width of (126 ± 31) nm, close to the initial simulated value. The error of 31 nm can be attributed by

some of the line profiles not being perpendicular to the wall.

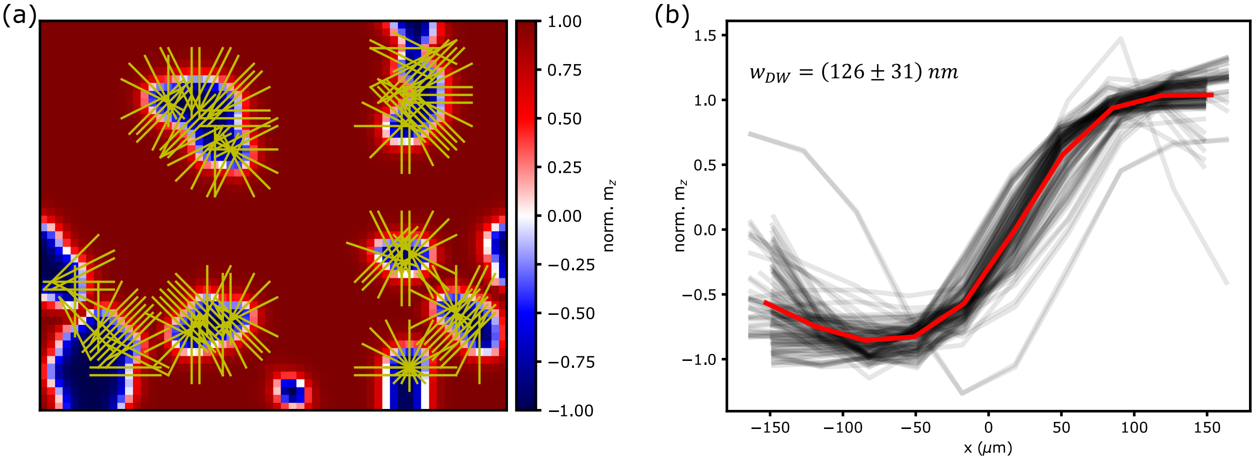

Figure S10: **Validating domain wall width extraction.** (a) Simulated m_z map based on the bubble domain morphology with a fixed wall width of 135 nm and the yellow line cuts to extract the width. (b) Resulting domain wall width of (126 ± 31) nm, the red curve is the average line profile.

S10 Robustness of domain wall analysis

In the domain wall analysis section we assumed fixed parameters for the NV-to-sample distance d_{NV} (80 nm) and the domain wall width w_{DW} (135 nm). Since both parameters are associated with a certain error, we show here that we can still reliably distinguish between Bloch and Néel walls even at the given error boundaries. To this end, we constructed a minimization matrix on the basis of the analysis explained in the main text and S7 and minimized the squared differences between experimentally obtained B_{NV}^{exp} and the simulated B_{NV}^{sim} with respect to the helicity ξ for a range of values for d_{NV} and w_{DW} covering the maximum and minimum bounds of our parameter errors. The resulting matrix is shown in Fig. S11, indicating an average helicity of $\pi/2$, hence suggesting that the resulting helicity is not greatly impacted by varying d_{NV} and w_{DW} in the parameter space associated with our data and analysis method. Specifically, with a spread of 70 nm to 100 nm for d_{NV} and of 60 nm to 200 nm for domain wall widths, the resultant average helicity is bounded between 0.4 and 0.68. Here, helicity holds a value from 0 to 2 which wraps around to 0, and perfectly

Néel being 0 (left) and 1 (right) while Bloch being 0.5 and 1.5. The resulting helicity bounds mean we can statistically conclude that domain walls observed are strongly Bloch and are reliably distinguishable from a perfectly Néel character.

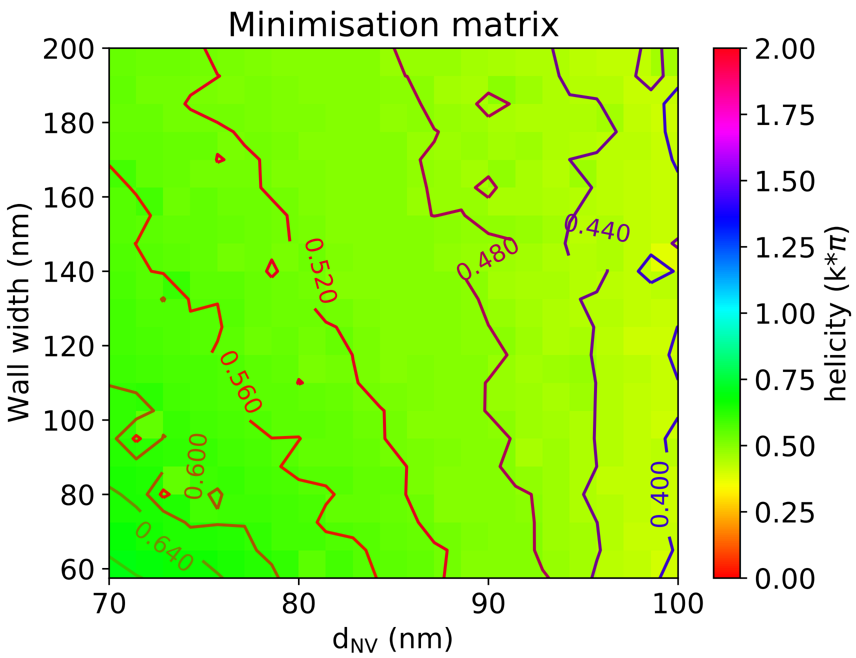

Figure S11: **Minimization matrix.** Minimization of the squared differences between experimentally obtained B_{NV}^{exp} and the simulated B_{NV}^{sim} with respect to the helicity ξ, as outlined in S8 for fixed $\mathrm{d_{NV}}$ and w_{DW}.

We provide additional visualizations of the difference in stray fields generated by bubble domains with typical pure Bloch, left Néel and right Néel domain walls as well as the error generated of each with the experimental data in Figs. S12, S13 and S14, respectively.

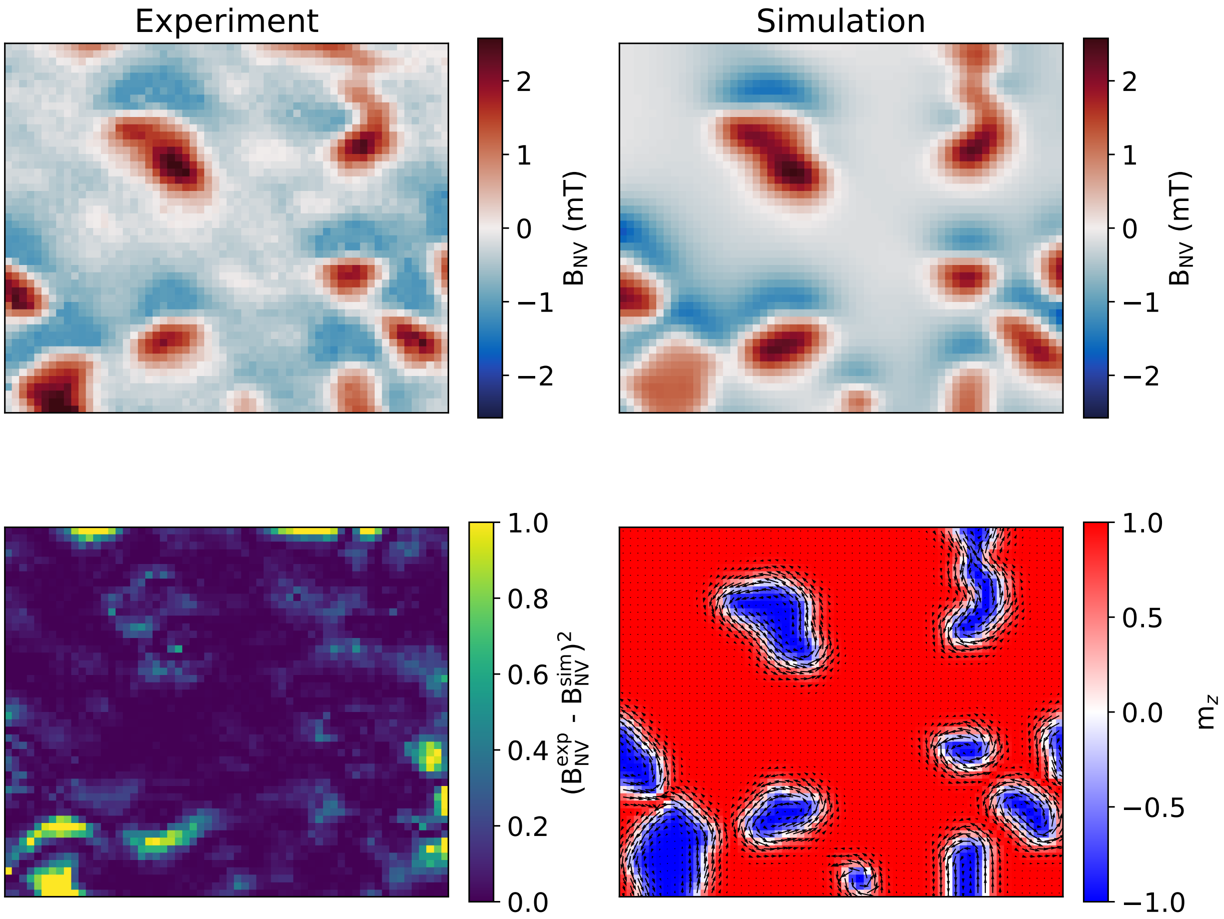

Figure S12: **Pure Bloch domain walls.** Top: Experimental stray field map compared to the simulated stray fields generated by domains with pure Bloch walls ($\xi = \pi/2$). Bottom: Squared error between experimental and simulated field maps and the magnetization used to generate the simulated stray fields.

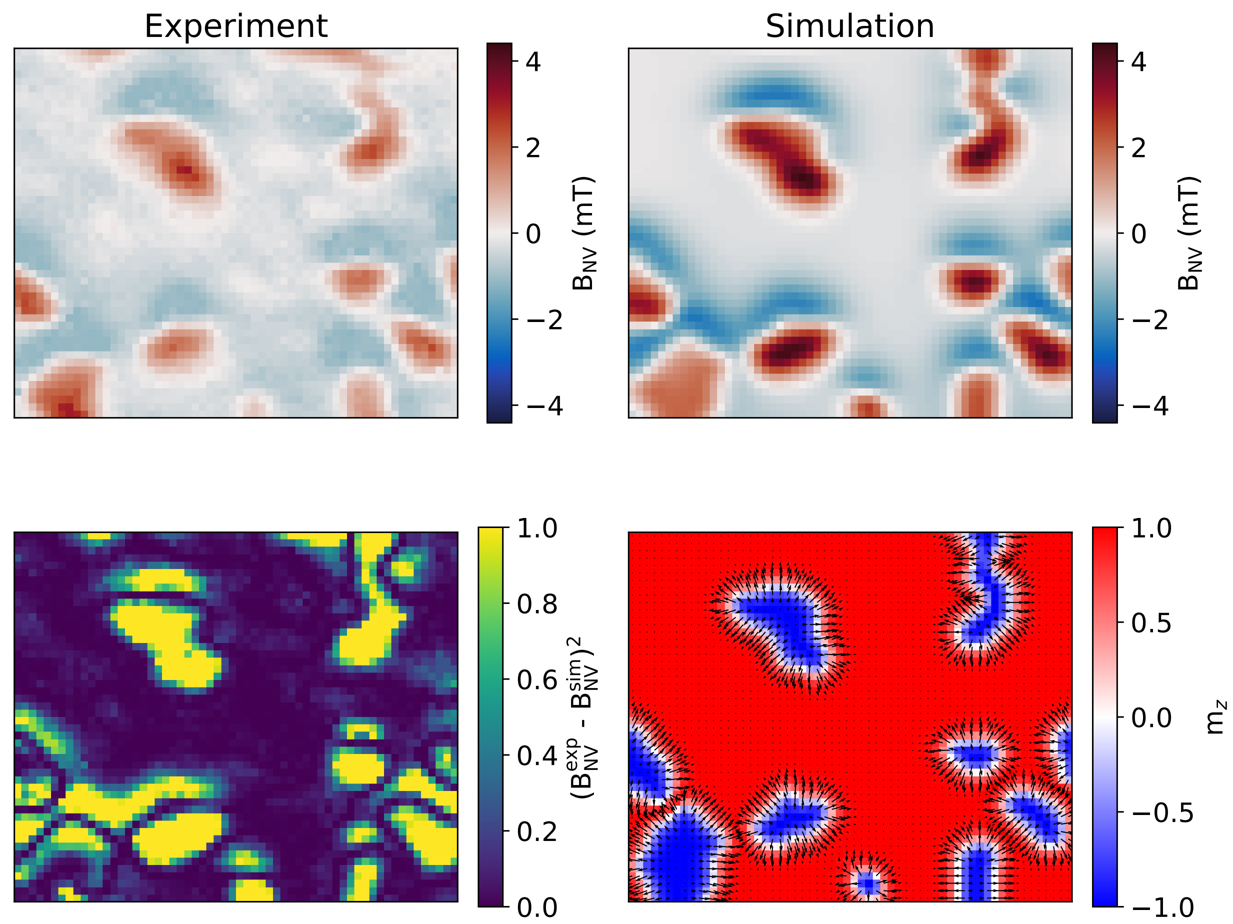

Figure S13: **Pure left Néel domain walls.** Top: Experimental stray field map compared to the simulated stray fields generated by domains with pure left Néel walls ($\xi = 0$). Bottom: Squared error between experimental and simulated field maps and the magnetization used to generate the simulated stray fields.

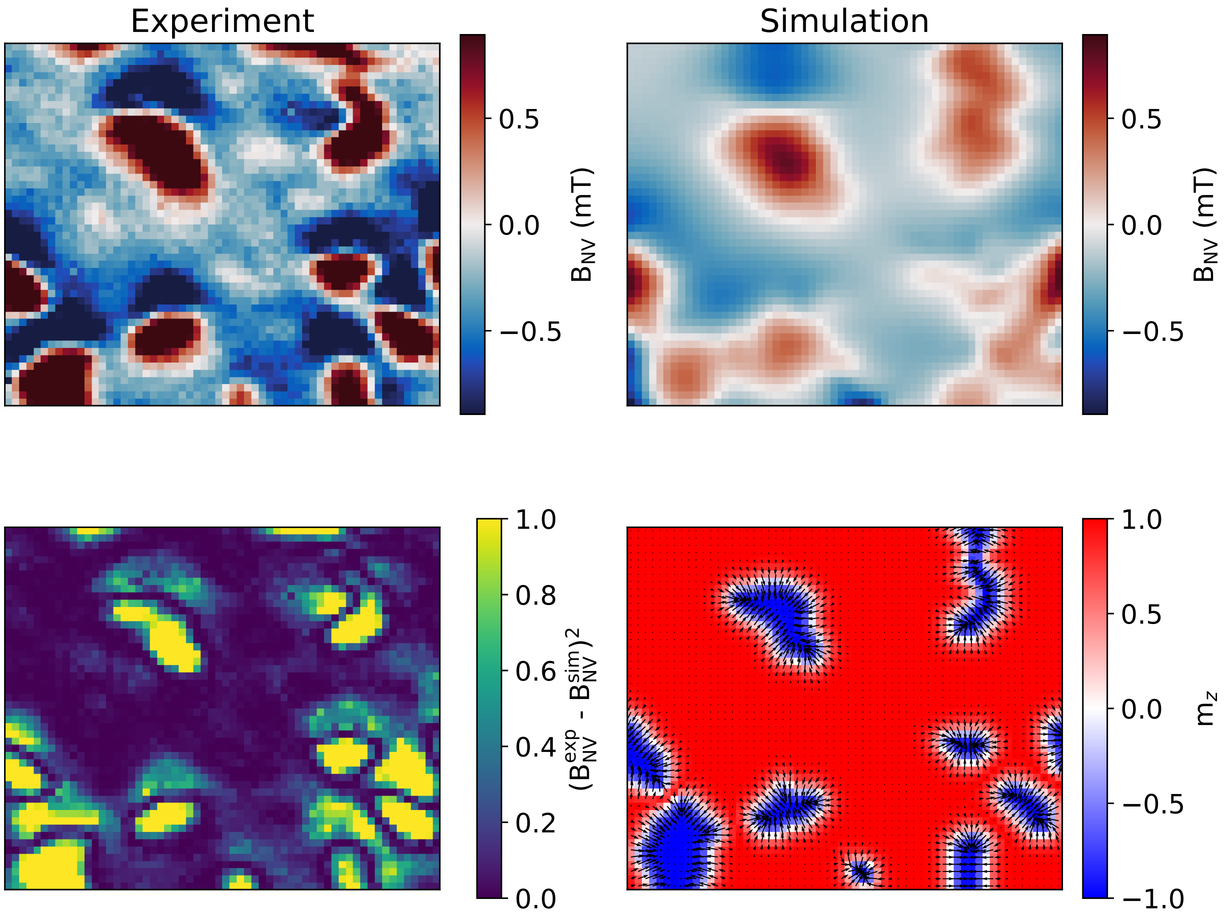

Figure S14: **Pure right Néel domain walls.** Top: Experimental stray field map compared to the simulated stray fields generated by domains with pure right Néel walls ($\xi = \pi$). Bottom: Squared error between experimental and simulated field maps and the magnetization used to generate the simulated stray fields.

S11 Reproducibility of domain formation

Here, we show a map of the same region acquired after a second cool-down, and compare it to the 3L bubble domain area shown in the main text. Most of the domains on the left map (second cool-down) are in the vicinity of, or overlapping with, the domains in the right map (first cool-down), strongly indicative of domain formation due to pinning sites.

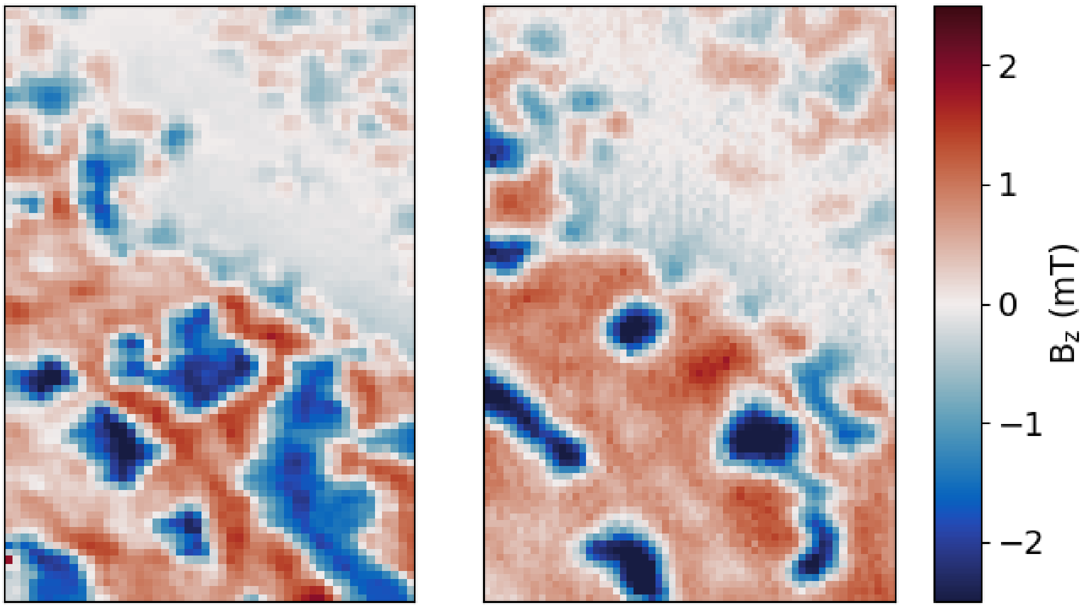

Figure S15: **Domain formation after second cooling.** Stray field map after second cool down (left) at the same location as after first cool-down (right) showing overlap of the down domains, strongly indicating domain formation due to pinning sites..

150 # S12 Additional data

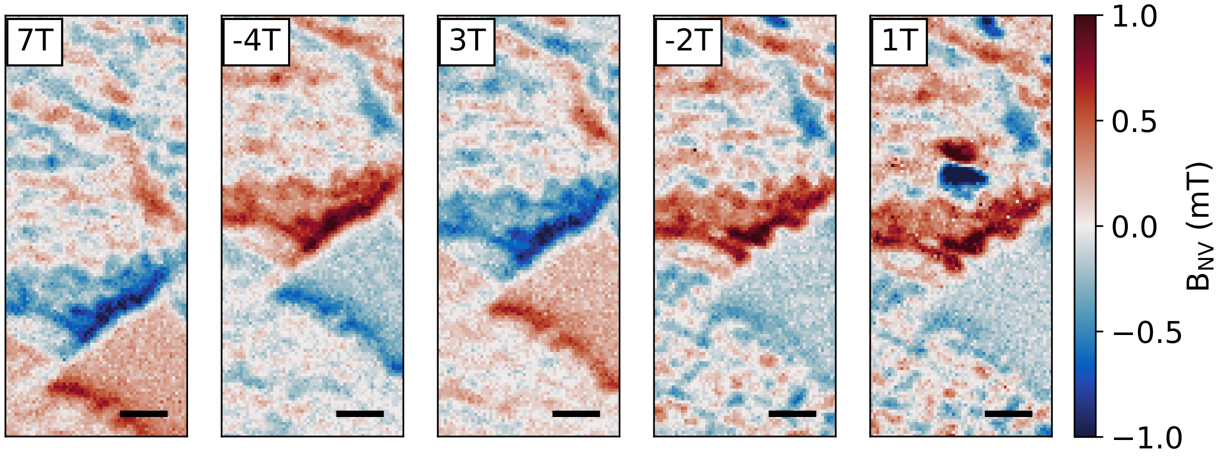

Figure S16: **Stray field maps.** Raw stray field maps used to reconstruct the m_z maps in the AC demagnetization section of Fig.2(b)-(e).

References

1. Dréau, A.; Lesik, M.; Rondin, L.; Spinicelli, P.; Arcizet, O.; Roch, J.-F.; Jacques, V. Avoiding power broadening in optically detected magnetic resonance of single NV defects for enhanced dc magnetic field sensitivity. *Phys. Rev. B* **2011**, *84*, 195204.

2. Rondin, L.; Tetienne, J.-P.; Hingant, T.; Roch, J.-F.; Maletinsky, P.; Jacques, V. Magnetometry with nitrogen-vacancy defects in diamond. *Rep. Prog. Phys.* **2014**, *77*, 056503.

3. Stefan, L.; Tan, A. K.; Vindolet, B.; Högen, M.; Thian, D.; Tan, H. K.; Rondin, L.; Knowles, H. S.; Roch, J.-F.; Soumyanarayanan, A.; Atatüre, M. Multiangle Reconstruction of Domain Morphology with All-Optical Diamond Magnetometry. *Phys. Rev. Appl.* **2021**, *16*, 014054.

4. Rohner, D.; Happacher, J.; Reiser, P.; Tschudin, M.; Tallaire, A.; Achard, J.; Shields, B.; Maletinsky, P. (111)-oriented, single crystal diamond tips for nanoscale scanning probe imaging of out-of-plane magnetic fields. *Appl. Phys. Lett.* **2019**, *115*, 192401.

5. Stefan, L. Scanning magnetometry with single-spin sensors. PhD thesis, University of Bristol, Bristol, UK, 2020.

6. Hingant, T.; Tetienne, J.-P.; Martínez, L.; Garcia, K.; Ravelosona, D.; Roch, J.-F.; Jacques, V. Measuring the magnetic moment density in patterned ultrathin ferromagnets with submicrometer resolution. *Phys. Rev. Appl.* **2015**, *4*, 014003.

7. Lima, E. A.; Weiss, B. P.; Baratchart, L.; Hardin, D. P.; Saff, E. B. Fast inversion of magnetic field maps of unidirectional planar geological magnetization. *J. Geophys. Res. Solid Earth* **2013**, *118*, 2723–2752.

8. Thiel, L.; Wang, Z.; Tschudin, M. A.; Rohner, D.; Gutiérrez-Lezama, I.; Ubrig, N.; Gibertini, M.; Giannini, E.; Morpurgo, A. F.; Maletinsky, P. Probing magnetism in 2D materials at the nanoscale with single-spin microscopy. *Science* **2019**, *364*, 973–976.

9. Sun, Q.-C.; Song, T.; Anderson, E.; Brunner, A.; Förster, J.; Shalomayeva, T.; Taniguchi, T.; Watanabe, K.; Gräfe, J.; Stöhr, R.; Xu, X.; Wrachtrup, J. Magnetic domains and domain wall pinning in atomically thin $CrBr_3$ revealed by nanoscale imaging. *Nat. Commun.* **2021**, *12*, 1989.

10. Broadway, D.; Lillie, S.; Scholten, S. C.; Rohner, D.; Dontschuk, N.; Maletinsky, P.; Tetienne, J.-P.; Hollenberg, L. Improved current density and magnetization reconstruction through vector magnetic field measurements. *Phys. Rev. Appl.* **2020**, *14*, 024076.
